# Assessment of behavioral changes associated with oral meloxicam administration at time of dehorning in calves using a remote triangulation device and accelerometers

**DOI:** 10.1186/1746-6148-8-48

**Published:** 2012-04-30

**Authors:** Miles E Theurer, Brad J White, Johann F Coetzee, Lily N Edwards, Ruby A Mosher, Charley A Cull

**Affiliations:** 1 Department of Diagnostic Medicine and Pathobiology, Kansas State University, Manhattan, USA; 2 Department of Clinical Sciences, Kansas State University, Manhattan, USA; 3 Current address: Department of Veterinary Diagnostic and Production Animal Medicine, Iowa State University, 1600 S. 16th Street, Ames, USA; 4 Current address: JBS USA, LLC, 1770 Promontory Circle, Greeley, USA

**Keywords:** Oral meloxicam, Behavior, Dehorn, Remote triangulation device

## Abstract

**Background:**

Dehorning is common in the cattle industry, and there is a need for research evaluating pain mitigation techniques. The objective of this study was to determine the effects of oral meloxicam, a non-steroidal anti-inflammatory, on cattle behavior post-dehorning by monitoring the percent of time spent standing, walking, and lying in specific locations within the pen using accelerometers and a remote triangulation device. Twelve calves approximately ten weeks of age were randomized into 2 treatment groups (meloxicam or control) in a complete block design by body weight. Six calves were orally administered 0.5 mg/kg meloxicam at the time of dehorning and six calves served as negative controls. All calves were dehorned using thermocautery and behavior of each calf was continuously monitored for 7 days after dehorning using accelerometers and a remote triangulation device. Accelerometers monitored lying behavior and the remote triangulation device was used to monitor each calf’s movement within the pen.

**Results:**

Analysis of behavioral data revealed significant interactions between treatment (meloxicam vs. control) and the number of days post dehorning. Calves that received meloxicam spent more time at the grain bunk on trial days 2 and 6 post-dehorning; spent more time lying down on days 1, 2, 3, and 4; and less time at the hay feeder on days 0 and 1 compared to the control group. Meloxicam calves tended to walk more at the beginning and end of the trial compared to the control group. By day 5, the meloxicam and control group exhibited similar behaviors.

**Conclusions:**

The noted behavioral changes provide evidence of differences associated with meloxicam administration. More studies need to be performed to evaluate the relationship of behavior monitoring and post-operative pain. To our knowledge this is the first published report demonstrating behavioral changes following dehorning using a remote triangulation device in conjunction with accelerometers.

## Background

Dehorning has been demonstrated to cause pain within 4 hours of the procedure [1] and identifying methods to mitigate pain is critical for animal welfare. Dehorning is a common practice performed in the beef and dairy industries and this procedure is often performed without administering analgesics. The percentage of beef calves born with horns has significantly decreased from 29.3% in 1992 to 12.4% in 2007 due to producers breeding for polled animals [2]. However, in 2007, 94% of the dairy operations in the United States still dehorned calves [3]. Producers dehorn cattle to prevent injuries to other animals, prevent injuries to animal handlers, and to make maneuvering and processing the cattle easier [4]. Dehorning is a common procedure and previous research has investigated potential adverse effects and methods to mitigate these deleterious impacts.

Animal behavior is frequently utilized to determine animal well-being or welfare [5], and previous research has examined calf behavior post-dehorning utilizing observational measurements of pain including head rubs, ear twitches, and tail flicks [6,7]. Meloxicam, a nonsteroidal anti-inflammatory drug, has been shown to reduce cortisol levels, heart rates, and respiratory rate in calves after dehorning [8]. Heinrich *et al.* (2010) [8] used accelerometers and determined that calves administered meloxicam were less active for 5 hours following dehorning compared to the placebo group. Heinrich *et al.* (2010) [8] also determined that meloxicam treated calves had less ear flicks and head shakes compared to the control calves after dehorning. Although these studies illustrate the effect of meloxicam in reducing some physiologic measures associated with discomfort, analgesics are not always included when painful procedures are conducted. The majority (92%) of producers dehorn calves at the same time as castration and only 21% administer analgesics while castrating [9]. In a survey of US veterinarians, respondents administered analgesics at the time of dehorning in 49% of beef and 63% of dairy calves less than six months of age [10]. Further research on the effects of potentially efficacious analgesic regimens may help provide validity to current industry pain mitigation recommendations.

Meloxicam is currently used as a canine and feline pain reliever for osteoarthritis [11], and McDougall *et al.* (2009) [12] have shown that meloxicam can be used effectively in the dairy industry to reduce the effects of mastitis and reduce culling rates. Meloxicam is an extralabel drug used under the Animal Medicinal Drug Use Clarification Act because there are no analgesic compounds approved for use in food animal production in the United States [13]. Meloxicam has been approved for use in several European countries as a single dose of 0.5 mg/kg of body weight with a withdrawal time of 15 days in meat and 5 days for milk [14]. Meloxicam has been shown to have peak availability in ruminant animals at 11.6 h and an average half-life of 27.5 hours when administered orally [15].

Objectively monitoring animal behavior may allow evaluation of analgesic effectiveness through quantifying related behavioral changes. Remote tracking devices have been used to analyze and evaluate the social activity of dairy cows [16,17], and similar technologies have been used to monitor calf behaviors following castration and inoculation with bovine respiratory disease [18-20]. Previous research indicates that spatial placement in a pen may be a good indicator of health and animal wellness by evaluating feeding and watering activity of calves and comparing it to illness [21,22]. These remote sensing technologies allow collection of objective data which can be used to compare behavior based on analgesic treatment without observer interference.

The objective of this study was to evaluate the effects of the oral administration of the nonsteroidal anti-inflammatory drug meloxicam on calf behavior patterns following the dehorning process. This study utilized objective measurements of activity and spatial placement within the pen using accelerometers and a remote triangulation device to evaluate calf behavior in the 7 day post-dehorning and the potential impact of analgesic regimens. Our hypothesis was that calves given meloxicam would spend more time lying down and in feeding areas (hay and grain) compared to the control group following dehorning. Conclusions from this study will be important in assisting animal health providers in selecting appropriate analgesic administration methods for calf dehorning procedures.

## Methods

All procedures were performed in accordance with the protocol approved by the Kansas State University Institutional Animal Care and Use Committee (Protocol # 2694). Calves were observed twice daily throughout the trial to ensure all that calves were healthy and had no adverse reactions to the treatment.

Prior to initial procedures and behavioral measurements, there was an 8 day acclimation period to allow the calves to adjust to the pen environment, group housing, and the remote sensing technology. Calves were dehorned following the acclimation period (trial day 0). Afterward, calves were released into the pen and behavior was continuously monitored for seven days using the accelerometer and spatial placement systems.

### Study calves and animal management

Twelve castrated male Holstein calves approximately ten weeks of age ranging in weight from 59 to 91 kg with an average of 72.1 kg were selected for this research trial. All calves were acquired through two sources and castrated a minimum of 2 weeks prior to trial initiation. Calves were individually housed and exposed to hay and grain beginning six weeks prior to trial initiation, but the calves were not weaned off of milk replacer completely until eight days prior to trial initiation.

Calves were randomly assigned to either the control (CON) or the meloxicam (MEL) group using a computer software program (Microsoft Office Excel 2007; Microsoft, Redmond, WA) in a complete blocked experimental design by calf weight. The randomization and blocking procedures produced groups of calves with initial average weight (+/− standard deviation) of 72.0 kg (+/− 11.2) for the CON calves and 71.2 kg (+/− 9.1) for the MEL group. The calves were group housed in a single pen at Kansas State University measuring 11.9 by 25.6 meters with an open face shed and fed a starter grain diet consisting of 18.0% protein, 3.2% crude fat, 7.0% crude fiber, 1.0% calcium, and 0.5% phosphorous (Herd Maker Supreme B90; Land O Lakes St. Paul, MN). For three days after arrival, calves were fed 0.9 kg per head of starter ration and had ad libitum access to hay and water. After dehorning (trial day 0), the calves had ad libitum access to grain, hay, and water. Grain was fed to the calves twice per day.

### Behavioral measurements using ubisense-A remote triangulation device

**Figure 1:**
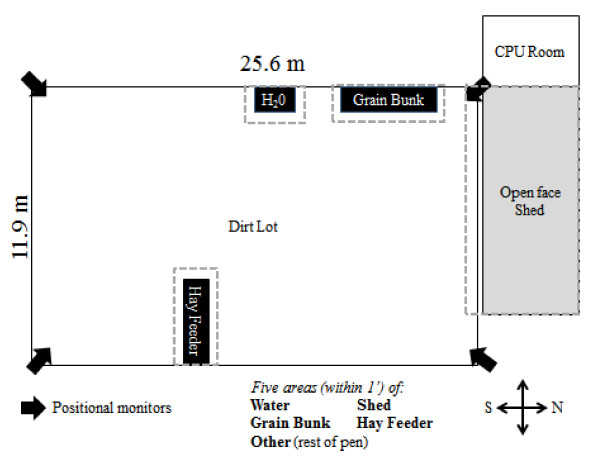
Model illustration of the pen layout and Ubisense sensor locations.

Three days prior to dehorning, all calves were affixed with Ubisense tags (Ubisense Series 7000 Compact Tag; Ubisense, Denver, CO) to monitor behavior and activity during the trial. Ubisense is a monitoring system used to evaluate position using a remote triangulation device using tags and sensors. The tags transmitted ultra-wideband radio pulses which were read by four sensors mounted at each corner of the pen. Properties of the tags featured a dual-radio architecture tag to determine precise location within the pen based on the previously mapped location of the grain bunk, hay feeder, shed, and water using the Ubisense system (Figure 1). The tags transmitted the location data to the sensors which forwarded data to a computer where the results were collected and stored [23]. The tags were placed on the calves three days prior to the dehorning to allow the calves to become accustomed to the monitoring devices. The compact design of the tags allowed us to attach each tag to a conventional ear tag button for each calf and be placed on top of the ear throughout the trial.

Calf positions were continuously recorded on a computer at the facility. Each data point was then classified to represent specific location in the pen using data mining software (Insightful Miner; Insightful Corporation Seattle, WA) by matching the X and Y coordinates of the tag to the known X and Y coordinates of the hay feeder, grain bunk, shed, and water. The data mining program classified the calf’s position at each time point based on the X and Y locations of the tag and within one foot of the grain bunk, hay feeder, water, shed, or remainder of the pen. The time each calf spent in all locations was aggregated by day. The Ubisense tags document a time stamp each time the sensors record the tag. The amount of time at each location was calculated by subtracting the time stamp recording from the previous reading and classifying the calf as being at the previous reading location. The distance each calf traveled was calculated by the Pythagorean Theorem based on changes in X and Y locations between time point collections. The total distance was aggregated by calf by day per day for analysis.

### Accelerometers

Three days prior to dehorning, calves were also equipped with commercially produced accelerometers (GP1 SENSR, Reference LLC, Elkader, IA) on the lateral aspect of the right hind leg proximal to the fetlock. Placement of the devices three days prior to dehorning was used as an acclimation period to allow the calves to become accustomed to the accelerometers; therefore, accelerometer data collected prior to dehorning were not used for analysis. The accelerometers were placed inside a freezer bag for water protection and then placed into a neoprene sleeve. The neoprene sleeve was equipped with Velcro and the sleeve was fastened securely to the leg. The accelerometers contain tri-axial measurements, have an axis range of ± 10 g, and record 100 samples per second [24]. The accelerometers were initialized with previously validated settings to measure cattle behavior [25] including five second recording intervals and recording X, Y, and Z acceleration, vector magnitude average, and vector magnitude maximum data. The average *g* values and vector magnitude average were calculated by summing the *g* and calf acceleration movement recordings and dividing by five second intervals. The vector magnitude max is the highest combined acceleration during the five second span.

At the conclusion of the study, the accelerometers and neoprene sleeves were removed from the calves and data were then downloaded using a USB cord connecting the accelerometers to a laptop computer. The data were processed using data mining software (Insightful Miner; Insightful Corporation Seattle, WA), and variables were classified using a previously validated system to determine the amount of time the calf spent standing, lying, and walking [25].

### Dehorning procedure

All calves were dehorned starting at 0800 hour on trial day 0. Dehorning order was based on convenience sampling (chute order) and procedures on all calves were completed in approximately one hour. The calves were dehorned using an electric hot-iron thermocautery (Rhinehart X30; Rhinehart Development Corporation, Spencerville, Indiana, USA) for twenty seconds on each horn. The dehorning instrument preheated for at least ten minutes to reach approximately 600°C before being used. Immediately after dehorning, the calves in the MEL group received 0.5 mg/kg of body weight of meloxicam (Unichem Pharmaceuticals, Rochelle Park, NJ) through an orogastric tube and then the tube was flushed with 50 cc of water. Following dehorning, calves in the CON group were administered a saline solution through an orogastric tube to serve as the negative control group. No calves received additional forms of analgesic therapy (e.g. local cornual nerve block) at the time of dehorning to allow discrete evaluation of the potential meloxicam effects. While the calves were in the processing chute the accelerometers were removed, data were downloaded and accelerometers were re-initialized, and re-attached to the calves.

### Statistical analysis

Data, from both the accelerometers and Ubisense tags, were summarized into the percent of time spent in respective postures and locations each hour within each trial day. The proportion of time spent in each location or posture for each hour were based on the number of data points in each category divided by the total number of known data points in that hour. All data collected during the acclimation period before dehorning were removed before analysis, and data prior to 1400 on day 0 (dehorning) were removed to avoid potential confounding of behavioral changes following dehorning with behavioral changes associated with processing and handling. Following day 0, any data that did not cover an entire 24 hour day (0800 to 0800) for a calf were removed before analysis to ensure the entire calf activity was recorded for all 24 hours due to calf activity varying throughout the day [26].

Data were transported into a commercial statistical software package (SAS 9.1.3, SAS Institute, Cary, NC) for descriptive and statistical analyses. A linear model was used to evaluate distance traveled and potential associations with trial day, treatment, and the interaction between these variables. The proportions of time spent in each location or posture (in seconds or minutes, respectively) were analyzed using logit models to test for associations of potential effects in a similar manner to previous work [19]. Model effects included treatment (MEL, CON), trial day, and the interaction between these two variables. Statistical models were constructed in a stepwise procedure by including all potential effects and removing non-significant (*P* > 0.05) effects until final model achieved. A first-order autoregressive correlation structure was defined to account for the repeated measures on calves over time in all analyses [27]. Type 3 likelihood-ratio statistics were used to test for associations of effects and comparisons with a *P* value < 0.05 were considered statistically significant. Potential differences between treatment groups within individual trial days were evaluated using t-tests.

## Results

All calves were evaluated twice daily and determined to be clinically healthy for the duration of the trial. Accelerometer data were unavailable from one calf in the CON group (trial days 1, 2, and 5) and another calf in the MEL group (trial days 1 and 2) due to mounting apparatus malfunctions. Data from the triangulation system (Ubisense) from one calf in the MEL group were omitted for the entire trial period due to a tag malfunction. Calf location was recorded by the triangulation system on average every 49 seconds and median value of 7 seconds between readings for each calf throughout the trial.

**Figure 2:**
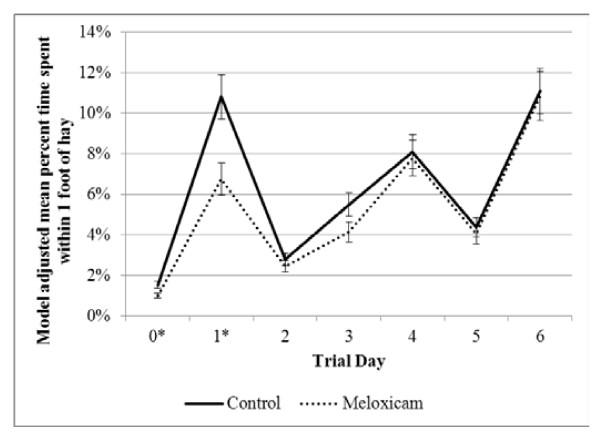
**Percent of time calves spent within 1 foot of the hay feeder.** Model representing the interaction (*P* = 0.03) of treatment (negative control and meloxicam calves orally administered 0.5 mg/kg meloxicam) and trial day (24 hours after procedure). Model included effects for trial day, treatment group (meloxicam or control), and the interaction between trial day and treatment group. An effect was also included to account for repeated measures on individual calves. * denotes significant differences (*P* < 0.05) between treatment groups within trial day.

**Figure 3:**
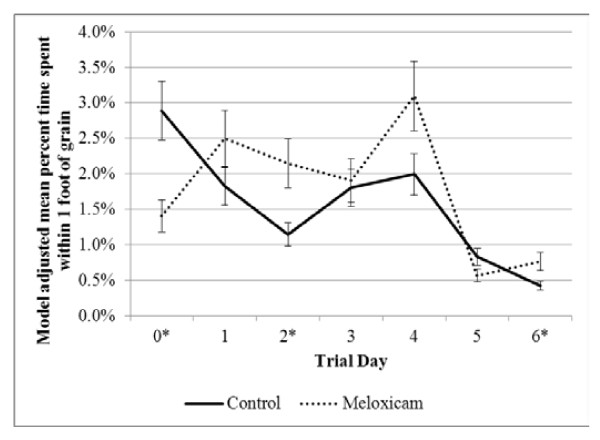
**Percent of time calves spent within 1 foot of the grain bunk.** Model representing the interaction (*P* = 0.03) of treatment (negative control and meloxicam calves orally administered 0.5 mg/kg meloxicam) and trial day (24 hours after procedure). Model included effects for trial day, treatment group (meloxicam or control), and the interaction between trial day and treatment group. An effect was also included to account for repeated measures on individual calves. * denotes significant differences (*P* < 0.05) between treatment groups within trial day.

**Figure 4:**
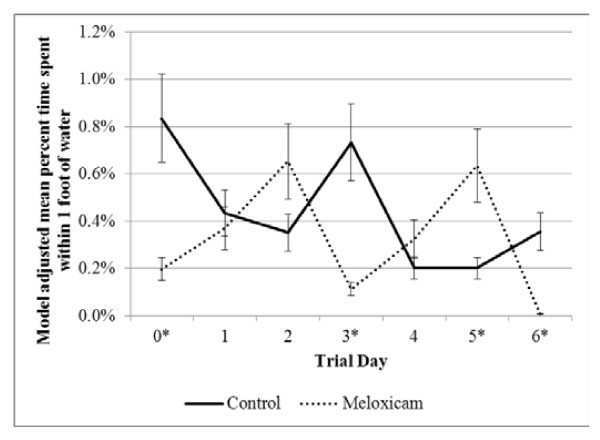
**Percent of time calves spent within 1 foot of the water.** Model representing the interaction (*P* = 0.03) of treatment (negative control and meloxicam calves orally administered 0.5 mg/kg meloxicam) and trial day (24 hours after procedure). Model included effects for trial day, treatment group (meloxicam or control), and the interaction between trial day and treatment group. An effect was also included to account for repeated measures on individual calves. * denotes significant differences (*P* < 0.05) between treatment groups within trial day.

The interaction between treatment group (MEL or CON) and trial day (0 through 7) was significantly (*P* < 0.05) associated with time spent within one foot of the hay feeder, grain bunk, water, shed, and other areas of the pen. Figure 2 illustrates that the amount of time the MEL calves spent at the hay feeder was lower on days 0 and 1 relative to the CON calves. The MEL group spent less time at the grain bunk on day 0 and then more time on days 2 and 6 compared to the CON calves (Figure 3). Both the CON and MEL groups displayed a variable amount of time spent next to the water; however, as a total percentage of each day, calves spent very little time next to the water (Figure 4). The MEL calves spent less time next to the water on days 0, 3, and 6 and more time on day 5 compared to the CON calves. There was no significant treatment group effect for distance traveled, but this variable was significantly (*P* < 0.05) associated with trial day (Table 1). Distance traveled was highest on trial days 3 and 4.

**Table 1:** **Model adjusted**^**1**^**least square mean daily distance traveled (24 hours after period) for all calves following dehorning on trial day 0**

	**Distance traveled**	
Trial Day	Meters	Standard Error
0	58.2	6.0
1	92.0	7.1
2	67.4	6.6
3	103.1	6.8
4	97.6	7.2
5	58.5	7.2
6	90.1	7.0

^1^ Model included effects for repeated measures on individual calves and fixed effect of trial day to begin when procedure performed. Treatment (MEL, CON) was not significantly (*P* > 0.05) associated with distantce traveled.

**Figure 5:**
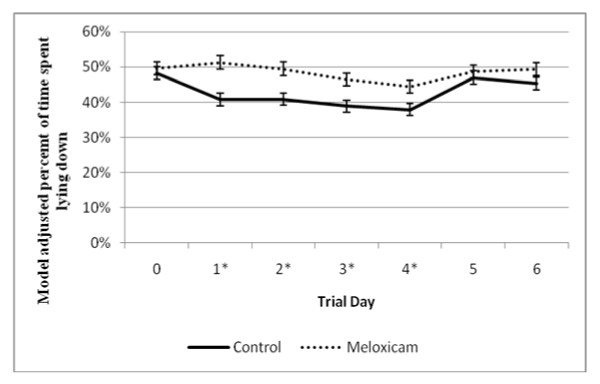
**Percent of time calves spent lying down.** Model representing the interaction (*P* < 0.01) of treatment (negative control and meloxicam calves orally administered 0.5 mg/kg meloxicam) and trial day (24 hours after procedure). Model included effects for trial day, treatment group (meloxicam or control), and the interaction between trial day and treatment group. An effect was also included to account for repeated measures on individual calves. * denotes significant differences (*P* < 0.05) between treatment groups within trial day.

**Figure 6:**
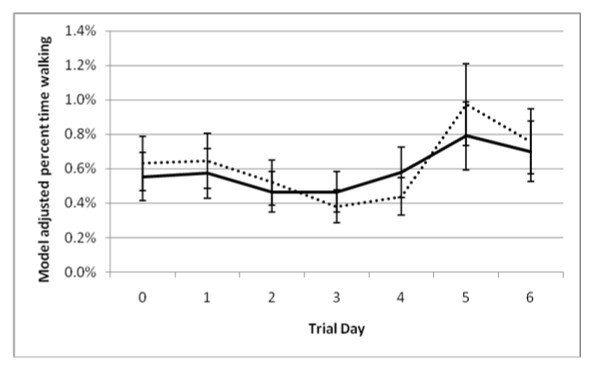
P**ercent of time calves spent walking.** Model representing the interaction (*P* < 0.01) of treatment (negative control and meloxicam calves orally administered 0.5 mg/kg meloxicam) and trial day (24 hours after procedure). Model included effects for trial day, treatment group (meloxicam or control), and the interaction between trial day and treatment group. An effect was also included to account for repeated measures on individual calves. * denotes significant differences (*P* < 0.05) between treatment groups within trial day.

Evaluation of the percent time lying, walking, and standing, as measured by accelerometers revealed a significant (*P* < 0.05) interaction between treatment group and trial day. The MEL calves spent more time lying down than the CON calves on days 0, 2, 3, and 4 (Figure 5). The amount of time spent walking as assessed by accelerometers also showed a significant interaction between treatment group and trial day (Figure 6).

## Discussion

These results show that meloxicam orally administered to calves at the time of dehorning had effects on time spent at specific locations in the pen and lying activity during the seven days post-dehorning in this study. The cattle behavioral patterns illustrated an interaction between treatment and trial day, providing evidence that the meloxicam treatment was associated with changes in calf behavior, but these changes did not last the entire 7 days post-dehorning monitoring period. Faulkner and Weary (2000) [28] determined that administering a nonsteroidal anti-inflammatory drug to dairy calves while dehorning changes head shaking and ear flicking behaviors, but the effect of nonsteroidal anti-inflammatory meloxicam on pen activity has not been well described. Heinrich *et al.* (2010) [8] measured the behavior differences in calves post-dehorning receiving meloxicam using measurements of head shakes and ear flicks.

Sowell *et al.* (1999, 1998) [29,30] concluded that morbid steers spent less time at the grain bunk compared to healthy steers in the feedlot. Their conclusions agree with the findings of this study that the MEL calves spent more time around the grain bunk on day 1 compared to the CON group (Figure 2). Calves that were administered MEL have shown to have a higher average daily gain ten days post-dehorning [31]. Our study may offer an explanation for this finding as calves in the MEL group spent more time in the vicinity of the grain bunk. Todd *et al.* (2010) [32] administered MEL to calves with diarrhea and determined that MEL treated calves gained body weight faster and consumed more feed than the CON calves. This agrees with our results showing that MEL may alleviate pain associated with dehorning which could in turn cause an increase in appetite.

Doherty *et al.* (2007) [33] determined no statistical difference in the amount of time spent eating and drinking between calves administered lidocaine and the saline control groups post dehorning. This differs from our current findings and could be attributed to the different mode of action of the drug administered. Doherty *et al.* (2007) [33] administered 2% and 5% formulations of the anesthetic lidocaine. Research has demonstrated the effects of 2% lidocaine lasting 81.8 minutes in cattle [34]. Coetzee *et al.* (2009) [15] determined that oral meloxicam has peak bioavailability at 11.6 hours and a half-life of 27.5 hours. The behavioral difference we observed related to grain bunk activity coincides with the time period when we expect the meloxicam to be most active (days 1 to 4). With meloxicam having longer lasting effects than lidocaine, this could affect behavior activity differently due to different analgesics administered. The difference between the Doherty *et al.* (2007) [33] study and our current study could also be partially attributed to the fact that they used visual observations and used 10 minute scan sampling with 144 observations per day for 3 days whereas we used 24 hour continuous surveillance for 7 days. On trial day 5, the MEL and CON groups both decreased the percent of time spent near the grain bunk to similar levels which may demonstrate the effects of meloxicam diminished.

In contrast to behavior at the grain bunk, the percent of times calves spent at the hay feeder was similar between treatment groups for all days except days 0 and 1 when calves in the CON group illustrated a marked increase in time at the hay feeder compared to MEL calves (Figure 1). The monitoring devices measure proximity to each feed area, not actual feed intake; therefore, the control calves may have been spending additional time at the hay feeder, but not actually eating. The wide diversity in the time spent at the water between the two groups provides some variability in the results and warrants further research. Although the percent time spent in the shed and other areas of the pen illustrated significant interactions between treatment group and trial day, there were no discernible patterns of behavioral change and no differences within trial days between treatment groups were identified.

The differences associated with the percent of time spent lying down between treatment group by trial day shows that meloxicam may be effective by increasing quiescence in calves (Figure 4). The meloxicam group spent a similar amount of time lying down throughout the trial whereas the CON group spent less time after dehorning and then reached similar levels to the MEL group on day 5. This lying activity would coincide of the effects of the analgesic wearing off on day 5. Mosher *et al.* (2010) [31] determined that calves that were administered meloxicam did not change lying behavior from pre to post-dehorning whereas within the placebo group lying activity decreased post-dehorning. Heinrich *et al.* (2010) [8] determined that calves administered MEL were less active for only 5 hours post-dehorning. This differs from our findings showing that calves administered MEL had increased lying activity for 5 days. This could be attributed to the fact that they had calves individually housed making activity monitoring difficult to perform and analyze and they administered lidocaine with intramuscular injection of meloxicam, whereas the calves in this trial were group housed and no local anesthetic was used.

Lying behavior can be associated with other painful events such as castration. González *et al.* (2009) [35] discovered that castration reduced time spent lying down. White *et al.* (2008) [19] determined that calves spent 82.2% time standing post-castration compared to only 37.9% pre-castration. This contradicts previous results where there were no significant differences in the percentage of time spent lying down between calves administered analgesic and the CON group [33]. This differs from our current findings and again could be attributed to the mode of action of the analgesic, response to pain, or the method of behavior observation.

Lying behavior may be a good indicator of animal well-being. Lying behavior has also been associated with animal morbidity [18,20]. An increase in amount of time standing could be related to pain. Hanzlicek *et al.* (2010) [20] determined that calves spent a greater proportion of time lying down after they were inoculated with *Mannheimia haemolytica* inflicting bovine respiratory disease. Hanzlicek *et al.* (2010) [18] also indicated that morbid animals spent more time lying down. This could be due to the depression associated with respiratory disease whereas dehorning is a more acutely stressful procedure. Treatment group was not associated with the percent time walking or the distance travelled. Research needs to be performed to determine the effect of analgesics on calves while dehorning to determine if behavior analysis is an adequate indicator for pain recognition. Further research also needs to be performed on the effect of meloxicam on different ages of calves.

Limitations of this trial include housing calves in a small pen environment and determining how behavior is related to pain and performance. Previous work has illustrated that calves displayed less activity and less sensitivity to pressure algometry following dehorning with meloxicam administration [8], and these findings support a link between measures of behavior and pain. Application of these results are limited to dehorning young calves, and it is important to note that all calves were dehorned in this trial. Some of the differences in behavior may be an effect of the meloxicam itself, but there have been studies performed with both dehorned and non-dehorned calves administered meloxicam that have shown effect of behavior changes beyond just the drug [28,36]. The addition of a treatment group that was not dehorned, yet received meloxicam, could have helped delineate the potential behavioral effect of meloxicam without a pain event. Additionally, there are other common pain mitigation techniques (e.g. cornual nerve block) that were not tested in this experiment, and they should be evaluated in subsequent work. The triangulation system did not record location data at pre-defined intervals, but rather signals were triggered by movement resulting in varied intervals between location data points. The median interval of 7 seconds between location readings supports that location was monitored frequently; however, long periods of inactivity may result in a larger interval between data points. An objective of the trial was to compare location patterns between treatment groups and cattle in both groups were monitored using tags with the same reporting criteria.

## Conclusions

Oral meloxicam can be administered to calves in a relatively easy way and have potential positive effects on calf behavioral changes post-dehorning. The increase in proportion of time lying down and around the grain bunk warrants more research to further analyze the physiological effects and efficiency of meloxicam administration achieved in animal production under field conditions. To the authors’ knowledge, this is the first report that utilized a remote triangulation device as a method of assessing animal behavior.

## Abbreviations

CON: Control; MEL: Meloxicam.

## Competing interests

The authors declare that they have no competing interest.

## Authors’ contributions

All authors were involved in the designing of the study. MET, RAM, and CAC all performed the study. MET and BJW analyzed the data. MET prepared the manuscript and all authors contributed to, read, and approved the final manuscript.
